# *Bifidobacterium infantis* supplementation versus placebo in early life to improve immunity in infants exposed to HIV: a protocol for a randomized trial

**DOI:** 10.1186/s12906-023-04208-0

**Published:** 2023-10-18

**Authors:** Anna-Ursula Happel, Lerato Rametse, Brandon Perumaul, Christian Diener, Sean M. Gibbons, Donald D. Nyangahu, Kirsten A. Donald, Clive Gray, Heather B. Jaspan

**Affiliations:** 1 Department of Pathology, University of Cape Town, Anzio Road, Observatory, Cape Town, 7925 South Africa; 2 Institute of Infectious Disease and Molecular Medicine, University of Cape Town, Anzio Road, Observatory, Cape Town, 7925 South Africa; 3 Institute for Systems Biology, Seattle, WA 98109 USA; 4 Department of Bioengineering, University of Washington, Seattle, WA 98195 USA; 5 Department of Genome Sciences, University of Washington, Seattle, WA 98195 USA; 6 eScience Institute, University of Washington, Seattle, WA 98195 USA; 7 Seattle Children’s Research Institute, 307 Westlake Ave. N, Seattle, WA 98109 USA; 8 Division of Developmental Paediatrics, Department of Paediatrics and Child Health, Red Cross War Memorial Children’s Hospital, University of Cape Town, Klipfontein Road Rondebosch, Cape Town, 7700 South Africa; 9 The Neuroscience Institute, University of Cape Town, Anzio Road, Observatory, Cape Town, 7925 South Africa; 10 Division of Molecular Biology and Human Genetics, Stellenbosch University, Francie Van Zijl Drive, Tygerberg, 7505 South Africa; 11 Department of Pediatrics, University of Washington, 1959 NE Pacific St, Seattle, WA 98195 USA; 12 Department of Global Health, University of Washington, 1510 San Juan Road NE, Seattle, WA 98195 USA

**Keywords:** HIV exposure, South Africa, Live biotherapeutic, Randomised controlled trial, Bifidobacterium

## Abstract

**Introduction:**

Infants who are born from mothers with HIV (infants who are HIV exposed but uninfected; iHEU) are at higher risk of morbidity and display multiple immune alterations compared to infants who are HIV-unexposed (iHU). Easily implementable strategies to improve immunity of iHEU, and possibly subsequent clinical health outcomes, are needed. iHEU have altered gut microbiome composition and bifidobacterial depletion, and relative abundance of *Bifidobacterium infantis* has been associated with immune ontogeny, including humoral and cellular vaccine responses. Therefore, we will assess microbiological and immunological phenotypes and clinical outcomes in a randomized, double-blinded trial of *B. infantis* Rosell®-33 versus placebo given during the first month of life in South African iHEU.

**Methods:**

This is a parallel, randomised, controlled trial. Two-hundred breastfed iHEU will be enrolled from the Khayelitsha Site B Midwife Obstetric Unit in Cape Town, South Africa and 1:1 randomised to receive 8 × 10^9^ CFU *B. infantis* Rosell®-33 daily or placebo for the first 4 weeks of life, starting on day 1–3 of life. Infants will be followed over 36 weeks with extensive collection of meta-data and samples. Primary outcomes include gut microbiome composition and diversity, intestinal inflammation and microbial translocation and cellular vaccine responses. Additional outcomes include biological (e.g. gut metabolome and T cell phenotypes) and clinical (e.g. growth and morbidity) outcome measures.

**Discussion:**

The results of this trial will provide evidence whether *B. infantis* supplementation during early life could improve health outcomes for iHEU.

**Ethics and dissemination:**

Approval for this study has been obtained from the ethics committees at the University of Cape Town (HREC Ref 697/2022) and Seattle Children’s Research Institute (STUDY00003679).

**Trial registration:**

Pan African Clinical Trials Registry Identifier: PACTR202301748714019. Clinical.trials.gov: NCT05923333.

Protocol Version: Version 1.8, dated 18 July 2023.

## Background

Globally, it is estimated that 1.6 million infants are born annually to mothers living with HIV. While implementation of successful prevention programs has reduced rates of perinatal HIV acquisition to as low as 1% [1], risk of morbidity and mortality in sub-Saharan African infants who are exposed to HIV but uninfected (iHEU) is still higher compared to infants who are HIV-unexposed (iHU) [2–4], even when accounting for maternal health and socioeconomic factors [3]. Skin, mucous membrane, and respiratory tract infections are more common in iHEU [2, 4], suggesting a more vulnerable immunological state contributing to increased morbidity. We and others have shown that iHEU display multiple immune alterations compared to iHU [5–10]. Humoral vaccine responses are different from iHU [7, 9], and cellular immunity may also be altered in iHEU, including responses to Bacille Calmette Guerin (BCG), a live, attenuated *Mycobacterium bovis* vaccine [5, 8]. iHEU have elevated immune activation, including heightened T cell and monocyte activation [6] and elevated inflammatory cytokine responses [10]. In addition, in multiple settings iHEU have demonstrated impaired growth [11, 12], even after adjusting for gestational age at birth [11], and poorer neurodevelopment [13, 14]. Since iHEU make up over 15% of infants in some parts of the world [15], easily implementable strategies, ideally using readily available interventions, to improve immunity of iHEU and subsequent short-and longer-term health outcomes, are urgently needed.

Early-life colonization of the infant’s mucosal surfaces plays a pivotal role in maturation of the immune system [16, 17]. The infant gut is colonized with bacteria during and after delivery [18–20], and the community composition of iHEU gut microbiota is altered longitudinally compared to iHU [21–24], with iHEU having lower abundances of *Bifidobacteria* during early life [23]. This is not surprising as women with HIV have decreased *Bifidobacteria* in their gut [25], and maternal gut microbiota is a strong determinant of infant gut microbiota [26–28].

Emerging data suggest that the gut microbiota is related to mucosal vaccine responsiveness in infants [29–31]. Furthermore, Huda et al*.* found that CD4 + T cell stimulation index in response to intradermal BCG vaccine at 15 weeks of age positively correlated with *B. infantis* abundance at 6 weeks of age, and an earlier studies showed that breastfeeding at time of vaccination was correlated with better cell-mediated immune response to BCG vaccine [32–34]. These observational studies suggest that *Bifidobacterium* abundance, and possibly specifically *B. infantis*, around the time of vaccination may improve cellular vaccine responses; however, the observed correlations do not establish causality.

Several interventional trials have tested the effect of *B. infantis* on infant health, including for diarrhoea treatment and necrotizing enterocolitis prevention [35–37]. *B. infantis* supplementation has shown beneficial effects on enteric inflammation, mucin degradation, and antibiotic resistance gene carriage [38–40]. Few studies have examined the effect of *B. infantis* supplementation on adaptive immunity and so far, none of these studies included iHEU. Deficient *Bifidobacteriaceae* in the gut has been associated with expanded populations of neutrophils, basophils, plasmablasts, and memory CD8 + T cells in infant blood, indicating both innate and adaptive immune activation [41]. Conversely, infants with abundant gut *Bifidobacteriaceae* had higher frequency of anti-inflammatory non-classical monocytes and CD39 + Tregs [41], a highly suppressive Treg subset [42]. *B. infantis* supplementation was shown to silence intestinal Th2 (allergenic) and Th17 (autoimmune) responses in Scandinavian infants [41]. Importantly, 3 weeks of daily *B. infantis* supplementation from postnatal day 7 to 28 was sufficient to alter the gut microbiota up to one year of age in iHU [43]. This was true even in infants who received antibiotics after *B. infantis* supplementation was completed, which is important because the World Health Organization (WHO) currently recommends iHEU receive co-trimoxazole prophylaxis, commencing at 6 weeks of age and continued until 6 weeks post-breastfeeding cessation.

Collectively, these data form the basis of our hypothesis that supplementation with *B. infantis* in early life will lead to beneficial changes in gut microbiome composition and function, improved gut mucosal integrity and a more stable immune homeostasis, resulting in decreased immune activation and improved antigen-specific T cell immunity in iHEU. We will conduct a randomized, placebo-controlled, quadruple-blinded trial of *B. infantis* Rosell®-33 given during the first month of life in 200 breastfed iHEU with regular follow-up over nine months and biological and clinical outcomes.

## Methods and design

### Objectives

The primary objectives of this study are to evaluate the effect of early-life *B. infantis* Rosell®-33 supplementation in iHEU on gut microbiome composition and diversity (objective 1), and markers of intestinal inflammation and microbial translocation (objective 2) at 4 weeks of life, and the effect on Th1 cytokine responses to BCG at 7 weeks of life (objective 3).

The secondary objectives of this study are to evaluate the effect of *B. infantis* Rosell®-33 supplementation on longitudinal succession of the gut microbiota composition, diversity and function, stool metabolome and T cell subset ontogeny during the first 9 months of life.

Exploratory objectives are to evaluate whether *B. infantis* Rosell®-33 supplementation improves infant growth, vaccine responses (including antibody titres) to other early childhood vaccines, all-cause morbidity or neurodevelopment during the first 9 months of life.

### Trial design

This is a two-arm, randomized, double-blinded, placebo-controlled trial of *B. infantis* Rosell®-33 supplementation in the first 4 weeks of life in 200 breastfed iHEU. iHEU will be block-randomized in a 1:1 ratio to receive ~ 8 × 10^9^ CFU of *B. infantis* Rosell®-33 versus placebo daily from postpartum day 3 to 28. Participants will be followed over 9 months. Assessment of infant outcomes and sample collection will be done at birth, day 10–14 and weeks 4, 7, 15, 24 and 36 of life (Table 1).

**Table 1:**
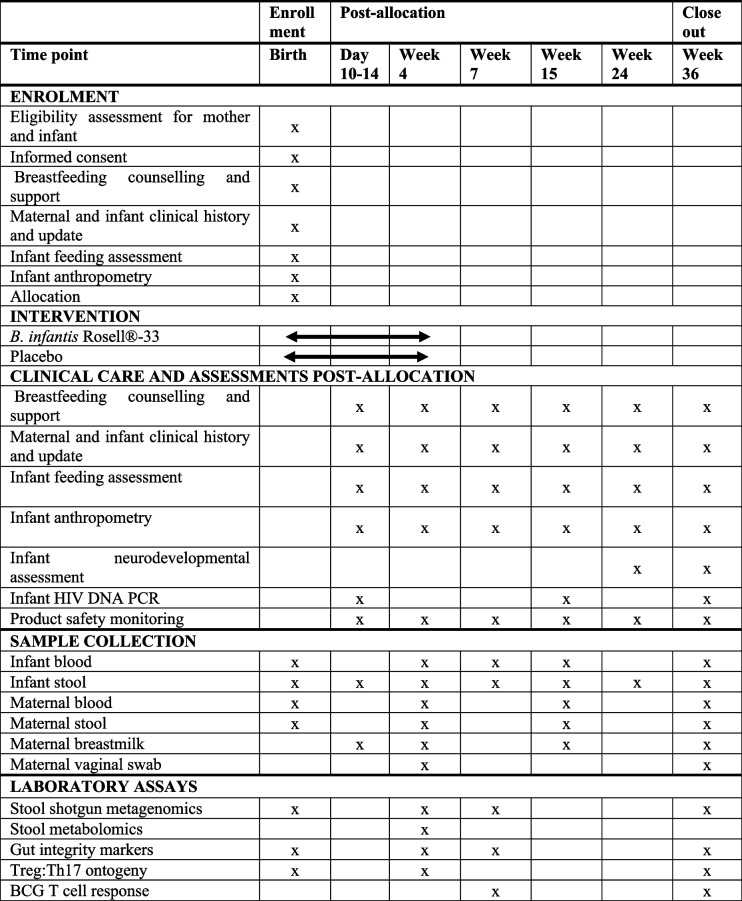
Schedule of enrolment, interventions and assessments

### Sample size

We propose to enrol 200 iHEU based on sample size calculation conducted for each of the three primary objectives.

- Objective 1: Sample size calculations were based on metagenomic data from D’Souza et al. (2020) [44], and on *B. infantis* copies in the stool collected at day 4–7 of life from infants in our current observational cohort from the same clinical site, where the mean *B. infantis* copy number was 3000 per 2.5 ng gDNA. The power analysis was based on a generalized linear regression approach, with a betabinomial error model for the dependent variable where varying effect sizes were injected to calculate effective power. The sample size of 100 iHEU per group is adequately powered to detect a difference of at least twofold for differential abundance testing and R^2^ values of 0.01 or higher in PERMANOVA analyses between iHEU administering *B. infanti*s Rosell®-33 versus those administering placebo, even after accounting for a lost-to-follow-up rate of 9% at week 36 (based on our observational cohort).
- Objective 2: Based on data from our observational cohort where the mean log intestinal fatty-acid binding protein (iFABP) plasma concentration in iHEU at day 4–7 was 0.454 ng/ml with a standard deviation of 0.164, we will have 80% power to detect a 20% difference in iFABP with *n* = 51 per group. Therefore, our proposed sample size of *n* = 100 per group will be ample to detect what we think is a meaningful difference.
- Objective 3: A sample size of 78 per group (total *n* = 156) will provide 80% power to detect a 45% increase in total net Th1 cytokine responses to BCG based on preliminary data in iHEU where the mean response was 0.78 and standard deviation 0.8, and the proposed sample size of 100 iHEU per arm accounts for a predicted loss-to-follow-up rate of 2.5% to 7 weeks, resulting in an expected sample size of *n* = 195 at week 7.

### Participants, intervention and outcomes

#### Study setting and recruitment

Mothers and infants will be recruited from the Khayelitsha (Site B) Midwife Obstetrics Unit in Cape Town, South Africa. Study staff will present information about the trial to all mothers in the waiting room of the antenatal clinic to sensitize women about the study prior to delivery. Identification of potential study participants will occur in the delivery ward. Routine Midwife Obstetrics Unit staff will alert study staff of an potential participant to approach. Those who could be eligible for the study and are interested will provide informed consent. Women will then be screened to confirm eligibility, and eligible participants will be enrolled in the study.

#### Eligibility criteria mother and infant

Maternal eligibility criteria are:

- Willing and able to provide signed and dated informed consent form
- 18 years of age or older
- Documented HIV seropositive and antiretroviral therapy initiated before the third trimester of pregnancy
- Planning on exclusively breastfeeding
- Planning on remaining in Cape Town until 9 months postpartum
- No severe illnesses, e.g. current tuberculosis (TB) or known household TB contact
- No eclampsia, or chronic disorder or medications (other than antiretrovirals and cotrimoxazole prophylaxis) that in the opinion of the investigator could alter immunity

Infant eligibility criteria are:

- Documented HIV seronegative at birth
- Born at term (completed at least 37 weeks of gestation), without pregnancy or delivery complications including birth asphyxia, seizures, sepsis, major congenital anomalies or congenital infections
- Birth weight > 2.4kgs
- No known contraindications to components of the interventional products
- Not taking additional probiotics or prebiotics
- Any condition that in the opinion of the investigator would make participation in the trial unsafe or otherwise interfere with the aims of the trial.

### Informed consent

Consent is performed by a trained study nurse or counsellor. Per South African Good Clinical Practice (GCP) guidelines, the mother’s literacy will be assessed, and study staff will assess basic understanding of the trial by asking her to repeat some of the information about the study. The mother will be consented in whichever language she feels most comfortable, isiXhosa or English, and consent forms will be available in either language. For illiterate participants, an impartial witness will sign to testify that the participant understood the consent.

### Intervention

The investigational product is *B. infantis* Rosell®-33, one of the three bacterial strains composing the commercial infant formula Probiokid®. The Food and Drug Administration (FDA) has classified these three strains, including *B. infantis* Rosell®-33, individually or as a blend, with the Generally Recognized as Safe status (GRAS notice 758, 2018), and clinical studies have shown the safety for use in infants younger than six months [45–47]. The formula has shown beneficial effects in children on immune maturation, anaemia, and reduction of common infections like diarrhoea and respiratory tract infections, as reviewed elsewhere [48]. Beneficial effects include long-term restructuring of the infant gut microbiota, an increased fecal anti-inflammatory signature, and significantly higher levels of stool and salivary sIgA [45–47]. As such, the strain alone has been already studied in a randomized, double-blind, placebo-controlled study, where *B. infantis* Rosell®-33 was deemed safe and well tolerated in 221 healthy, three- to twelve-months-old infants [46]. A post-hoc analysis based on a sample of infants from the Manzano 2017 study showed *B. infantis* Rosell®-33 maintained a infant-like microbiome profile rich in bacteria able to digest lactose such as *Bifidobacteria*, regardless of any influencing factors such as diet and birth mode [45]. In contrast, in the placebo group, there was significant increase in common constituents of healthy adult gut microbiota, and fewer *Bifidobacteria* (most specifically *Bifidobacterium bifidum* and *Bifidobacterium breve*).

Health Canada has recognized that *Bifidobacterium infantis* Rosell®-33 can help formula-fed babies develop a similar microbiota as to breast-fed infants.

For this trial, Lallemand Health Solutions will manufacture the investigational product containing 8 × 10^9^ CFU *B. infantis* Rosell®-33 per dose (single microbial active ingredient) and carrier material (maltodextrin), as well as the placebo (containing all materials besides *B. infantis* Rosell®-33). Mothers will be provided with two 14-day supplies of identical looking 1 g sachets containing either *B. infantis* Rosell®-33 or the placebo powder at enrolment and the first follow-up visit, both of which can be stored at room temperature. Mothers will be requested to pour one sachet (containing the investigational product or placebo) into a provided sterile cup daily, add 2 mL of expressed breastmilk, mix the powder and breastmilk, and then cup-feed her infant immediately. If, for any reason, the mother is unable to produce any/enough breastmilk during the intervention phase and is therefore using infant formula, she will be advised to mix the sachet with formula.

### Assignment of interventions and unblinding

Enrolled iHEU will be block-randomized in a 1:1 ratio to receive 8 × 10^9^ CFU of *B. infantis* Rosell®-33 or placebo daily from postpartum day 1–3 to 28–30. Randomization will be done by a statistician and the confidential randomization list will be provided to the pharmacist. The intervention and placebo products will be packaged in identical sachets with identical labelling. Participants, care providers, investigators and outcome assessors will be blinded to the participant’s allocated intervention.

Unblinding will occur if there is concern raised by the investigators or other clinical care providers, and they believe that unblinding would change the care provided. Unblinding might be requested from the human ethics committees, Data Safety and Monitoring Board (DSMB) or the sponsor. Unintentional unblinding will be recorded as study deviation.

### Adherence monitoring

Adherence will be encouraged and assessed using three measures: (i) self-report with daily record of administration using diaries and 1-day, 7-day and 2- or 4-week recall at the Day 10–14 and week 4 visits, respectively, (ii) return of empty sachets, (iii) and *B. infantis* Rosell®-33 Real-Time qPCR to assess absolute abundance in infant stool. We will triangulate self-report, sachet count and absolute *B. infantis* Rosell®-33 abundance in infant stool to assess adherence.

### Use of concomitant medication during the trial

Concomitant medications can be prescribed to infants as per the standard of care as necessary and will be recorded. However, mothers will be asked to not give their infants non-study probiotics or prebiotics during the study period.

### Participant timeline, data and sample collection

Participants will be followed from birth to 9 months of life, as outlined in the schedule of events (Table 1). Data and sample collection will be done by clinical staff. Upon enrolment, infants will be randomized to receive *B. infantis* Rosell®-33 or placebo for the first 4 weeks of life, starting on day 1–3 of life. Follow-up visits will be conducted at 10–14 days (for safety assessment and adherence monitoring and support), week 4 (end of the intervention), and weeks 7, 15, 24 and 36 of life, with adverse event solicitation, extensive questionnaires, anthropometrics, examinations, and sample collection performed (Table 1). Participant retention will be promoted through regular phone calls and messages. Maternal CD4 count and HIV viral load and infant HIV PCR test results will be accessed by the study coordinator from the National Health Laboratory Service password-protected web-based interface. An additional HIV DNA PCR will be performed at the Day 10–14 visit to ensure no missed HIV transmissions during labour. Any infant with a positive HIV DNA PCR will discontinue product. Maternal physical examination will include height, weight, and blood pressure. Infant physical exam will include an assessment of birth defects, anterior fontanelle, palette, heart sounds, abdominal masses, hip click, external genitalia, and sacral dimple at enrolment. Thereafter, length, weight and head circumference will be measured at each visit and a targeted exam for any symptoms. Infant health status will be assessed at each visit by interview and medical folder review using standardized forms. Detailed feeding questionnaires are used that have been validated in our and other similar settings [49]. At each visit, infant stool is collected taking care to avoid the diaper, placed at -20 °C, and transferred to the laboratory within 6 h to be stored at -80 °C. Infant blood will be collected at enrolment, week 4, 7, 15 and 36 and used for whole blood assay or PBMC will be extracted and stored on LN_2_ and plasma stored at -80 °C. Maternal blood, stool, vaginal swabs and breastmilk will be collected at week 4, 15 and 36 and biobanked for future studies (Table 1).

### Outcomes and statistical methods

#### Primary outcomes

Analyses will follow intention-to-treat (ITT) principles comparing the two randomised groups.

1. We will compare Alpha (Shannon) and Beta (Bray Curtis and UniFrac) diversity metrics, calculated from shotgun metagenomic sequencing data for each infant stool sample, between treatment arms at 4 weeks. Cross-sectional abundance differences for taxa and functional genes will be assessed using DeSeq2 (negative-binomial) [50] or Corncob (beta-binomial) [51] regression models, with the Benjamini–Hochberg multiple-test correction on the resulting *p*-values (to control the false-discovery-rate (FDR)) [52]. Regressions will be adjusted for confounders, like sex, weight, and feeding mode.
2. Markers of intestinal inflammation and microbial translocation at baseline, weeks 4, 7 and 36: We will compare markers of intestinal inflammation and microbial translocation (Lipocalin-2 (Lcn-2), soluble CD163 (sCD163), iFABP and lipopolysaccharide binding protein (LBP) measured by ELISA in infant plasma) cross-sectionally at each time point between groups using Mann–Whitney U tests. We will correlate absolute concentrations of *B. infantis* in the fecal microbiome with these markers. We will use a mixed-effects regression (MER) modeling approach to account for repeated measurements and for relevant covariates like feeding status and sex.
3. BCG-specific total net Th1 cytokine producing cells at 7 weeks of life: We will compare frequencies of total net cytokine producing cells in response to stimulation with BCG between arms at weeks 7 using linear regression after adjusting for confounders, like sex, weight, and feeding mode. Additionally, T cell response data will be analyzed by COMPASS [53] to compute a ‘functionality score’ or ‘polyfunctionality score’. This analysis will be a regression model testing the association between functionality or polyfunctionality scores and iHEU (*B. infantis* versus placebo). We will perform two-sided hypothesis testing with alpha = 0.05. If the model shows a significant result (*p* < 0.05), we will perform a series of univariate analyses in which we test for an association between a particular functional profile and arm. Those profiles identified as significantly associated will be incorporated into a multivariate linear regression model, which will include two-way interaction terms. This will allow us to create a model that includes the cytokine-producing profiles that are most associated with the intervention. We will test the classification accuracy of this model using receiver-operative curve (ROC) analysis. In all models, we will include sex, birth weight, and feeding mode as possible confounders.

#### Secondary outcomes

1. Longitudinal succession in gut microbiota composition, diversity and function during the first 9 months of life by randomisation arm: Longitudinal multi-omic variation will be visualized using PCoA and tSNE plots [54]. Significant cross-sectional differences in multi-omic profiles will be assessed using PERMANOVA. To integrate longitudinal data and control for confounders, we will build MER models that incorporate repeated measures (random effects) and time-invariant covariates (fixed effects).
2. Stool metabolome at 4 weeks of age life by randomisation arm: We will generate semi-targeted stool metabolomics data, which will be used to validate metagenome-constrained community-scale metabolic modelling of the gut microbiota, using the MICOM platform [55], which will be used to estimate metabolomic fluxes for other time points that lack direct metabolomic measurements. For cross-sectional metabolite differential abundance analyses, we will use generalized linear regression (continuous dependent variable) and logistic regression (Boolean dependent variable), with an FDR correction on the *p*-values, adjusting for confounders, like sex, weight, and feeding mode.
3. T cell subsets at baseline, weeks 4 and 36 weeks of life by randomisation arm: We will compare T cell subsets frequencies cross-sectionally between iHEU administering *B. infantis* Rosell®-33 versus placebo using Mann–Whitney U tests, and correlate these data with absolute concentrations of *B. infantis* in the gut microbiota. We will utilize multidimensional scaling of markers expressed on both CD4 + and CD8 + T cells using the metaMDS function and PERMANOVA from the vegan R package [56] to identify multivariate statistical differences in immune markers between treatment groups, using the “adonis” function from the vegan R package [56]. Unsupervised cell population identification will be performed using self-organizing maps and hierarchical clustering as implemented in the FlowSOM and metaclustering R packages [57]. Finally, we will use a MER modeling approach mentioned above to account for repeated measurements and for relevant covariates like feeding status, sex, and gestational age at birth.

#### Exploratory outcomes

1. Presence of *B. infantis* Rosell®-33 in iHEU stool: We will use Mann–Whitney U tests for analyzing absolute abundances of *B. infantis* Rosell®-33, assessed by qPCR, across treatment groups at each time point, with an FDR correction for multiple comparisons.
2. Infant growth—length for age Z scores (LAZ) at 36 weeks of age: LAZ scores will be generated using Intergrowth-21st software, which adjust for infant gestational age at birth and infant sex [58]. LAZ will be binarized into adequate (LAZ ≥ -1) and inadequate (LAZ < -1) and compared between arms. LAZ as a continuous variable will additionally be used in penalized linear regression models between *B. infantis* abundance will while adjusting for feeding. Finally, we will compare the prevalence of stunting at 36 weeks of age between groups using Chi-squared tests.
3. All-cause infectious morbidity: We will abstract outcome data related to infectious morbidity from participant’s health records throughout the study period and quantify and compare occurrence of infectious morbidity outcomes between randomisation arms. The proportion of infants with at least one all-cause infectious morbidity event will be calculated and compared by randomisation group using a Fisher’s exact test. Univariate logistic regression models will be used to compare risk of infectious morbidity by randomisation group, and multivariate logistic regression models will be used to adjust for confounders including infant birth weight and gestational age at birth.
4. Neurodevelopment: Neurodevelopment will be assessed comprehensively at weeks 24 and 36 of life, e.g. using the Bayley Scales of Infant and Toddler Development, and developmental scores will be compared by randomisation group.

### Additional analyses

In addition to the ITT analyses outlined above, we will conduct modified ITT (mITT) analyses, including only participants who took at least one week of study drug and per protocol analyses (including only participants who took all doses of study drug and reached the primary endpoint time points).

### Interim analyses

No interim analysis is planned. An interim analysis will only be conducted if determined necessary by the Data Safety Monitoring Committee (DSMB) to assess safety concerns or study futility based upon accumulating data.

### Data management

All participants will be assigned a unique number. The electronic REDCap database will be used to document consent electronically, and to directly record information about participant demographics, questionnaire-based information, clinical procedures, and laboratory tests that are conducted locally. Only de-identified data will be shared with collaborators via the final dataset.

### Protection of participant confidentiality

All study staff members will sign a pledge of confidentiality. All data will be kept confidential, coded, and kept under lock and key. Databases will be password protected and will remain as such. Results of microbiological and immunological testing will not be made available to the participants.

## Ethics, oversight, monitoring and dissemination

### Ethics

This protocol has been reviewed and approved by Research Ethics Committee from the University of Cape Town (HREC ref 697/ 2022) and Seattle Children’s Research Institute (STUDY00003679). If required, protocol amendments will be submitted to the regulatory bodies in line with intuitional and regulatory guidelines and participants will be informed. No fault compensation for participants will be provided by the University of Cape Town if necessary.

### Composition of the data monitoring committee, its role and reporting structure

The DSMB consists of a public health/paediatric clinical epidemiologist, and paediatricians and clinical researchers from Africa and the United States. Membership of the DSMB is independent from the sponsor and none of the members declared any conflict of interest. In addition, an external monitor has been appointed to monitor the trial conduct.

### Adverse event (AE) reporting and harm

For the purposes of this study, AEs include any new events absent at baseline, or events that were present during the baseline visit which increased in severity. Examples of AEs include but are not limited to the following: a clinical event such as bloody stools, vomiting, constipation, colic or irritability, fever. Adverse effects and the severity of clinical symptoms will be scored using the DAIDS Table for Grading the Severity of Adult and Pediatric AEs [59]. All related and unrelated AEs will be collected and reported in the pre-specified timelines to the DSMB, ethical and regulatory bodies.

### Dissemination

Throughout the study, we will engage in tailored community-engagement activities, including consultation with the long-standing Khayelitsha Health Forum. We will disseminate study results first to study participants, stakeholders including the Khayelitsha Health Forum and community representatives, then the communities at large. The results will be made freely available to the greater scientific community through publications in peer-reviewed international journals. Alongside, the full protocol, statistical code and data will be made publicly available. Authorship will be determined in line with ICMJE guidelines.

### Trial status

Recruitment started in August 2023.

## Discussion

In this manuscript we describe the protocol for a randomized trial of *B. infantis* supplementation versus placebo in early life to improve immunity in infants exposed to HIV.

Determining whether *B. infantis* Rosell®-33, a readily available intervention, is effective in improving gut health, inflammation, and immunity in iHEU, a growing and vulnerable pediatric population, could result in improved clinical management and health outcomes of iHEU. This is highly relevant for sub-Saharan Africa, where up to 30% of infants are exposed to HIV, as the knowledge gained by this project has the potential to lead to interventions to mitigate poor immunity and associated morbidity and mortality of iHEU.

## Data Availability

Data sharing is not applicable to this article as no datasets have yet been generated or analysed.

## Abbreviations

AE: Adverse event
BCG: Bacille Calmette Guerin
DSMB: Data Safety and Monitoring Board
GCP: Good Clinical Practice
GRAS: Generally Recognized as Safe status
FDA: Food and Drug Administration
FDR: False-discovery-rate
HREC: Human Research Ethics Committee
iFABP: Intestinal fatty-acid binding protein
iHEU: Infants who are HIV exposed but uninfected
iHU: Infants who are HIV-unexposed
ITT: Intention-to-treat
LAZ: Length for age Z scores
LBP: Lipopolysaccharide binding protein
Lcn-2: Lipocalin-2
MER: Mixed-effects regression
mITT: Modified ITT
ROC: Receiver-operative curve
sCD163: Soluble CD163
TB: Tuberculosis
WHO: World Health Organization
